# A triclinic polymorph of dichlorido(2-{[2-(isopropyl­ammonio)­eth­yl]imino­methyl-κ*N*}-5-meth­oxy­phenolato-κ*O* ^1^)zinc

**DOI:** 10.1107/S1600536812014341

**Published:** 2012-04-13

**Authors:** Ai-Tian Pei

**Affiliations:** a Zibo Vocational Institute, Zibo 255314, People’s Republic of China

## Abstract

The title compound, [ZnCl_2_(C_13_H_20_N_2_O_2_)], was first reported in the monoclinic space group *P*2_1_/*n* [Han *et al.* (2010 ▶). *Acta Cryst.* E**66**, m469]. This investigation reveals a triclinic polymorph in the space group *P*-1 with an asymmetric unit that contains two independent mol­ecules of the mononuclear zinc(II) complex. In each mol­ecule, the Zn^II^ atoms are coordinated in a bidentate fashion by the phenolate O and imine N atoms of the Schiff base ligand. Two Cl^−^ anions complete the tetra­hedral coordination in each case. The most obvious difference between the two forms is that the Zn—*L* (*L* = O, N, Cl) bond lengths in both unique mol­ecules are longer than those found in the monoclinic polymorph, or indeed in other similar complexes. In the crystal, mol­ecules are linked through N—H⋯O and N—H⋯Cl hydrogen bonds, forming chains along the *b* axis.

## Related literature

For the structures of zinc complexes with Schiff base ligands, see: Munro *et al.* (2009 ▶); Granifo *et al.* (2006 ▶). For a monoclinic polymorph of the title compound in the space group *P*2_1_/*n*, see: Han *et al.* (2010 ▶). For bond lengths in related zinc complexes, see: Ali *et al.* (2008 ▶); Zhu (2008 ▶); Wang (2007 ▶). 
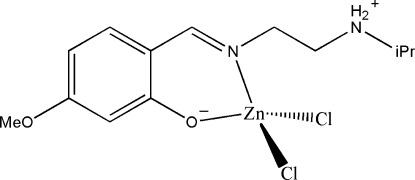


## Experimental

### 

#### Crystal data

[ZnCl_2_(C_13_H_20_N_2_O_2_)]
*M* *_r_* = 372.58
Triclinic, 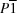

*a* = 6.491 (3) Å
*b* = 12.351 (2) Å
*c* = 22.803 (3) Å
α = 90.707 (2)°
β = 96.201 (2)°
γ = 90.660 (2)°
*V* = 1817.1 (9) Å^3^
*Z* = 4
Mo *K*α radiation
μ = 1.65 mm^−1^
*T* = 298 K
0.13 × 0.10 × 0.08 mm

#### Data collection

Bruker SMART CCD area-detector diffractometer
Absorption correction: multi-scan (*SADABS*; Sheldrick, 2004 ▶) *T*_min_ = 0.814, *T*_max_ = 0.880
8230 measured reflections
5775 independent reflections
4093 reflections with *I* > 2σ(*I*)
*R*_int_ = 0.027

#### Refinement

*R*[*F* ^2^ > 2σ(*F* ^2^)] = 0.055
*wR*(*F* ^2^) = 0.181
*S* = 1.08
5775 reflections
367 parameters
H-atom parameters constrained
Δρ_max_ = 0.63 e Å^−3^
Δρ_min_ = −0.57 e Å^−3^

### 

Data collection: *SMART* (Bruker, 2001 ▶); cell refinement: *SAINT* (Bruker, 2001 ▶); data reduction: *SAINT*; program(s) used to solve structure: *SHELXTL* (Sheldrick, 2008 ▶); program(s) used to refine structure: *SHELXTL*; molecular graphics: *SHELXTL*; software used to prepare material for publication: *SHELXTL* and local programs.

## Supplementary Material

Crystal structure: contains datablock(s) I, global. DOI: 10.1107/S1600536812014341/sj5230sup1.cif

Structure factors: contains datablock(s) I. DOI: 10.1107/S1600536812014341/sj5230Isup2.hkl

Additional supplementary materials:  crystallographic information; 3D view; checkCIF report

## Figures and Tables

**Table 1:** Selected bond lengths (Å)

Zn1—O1	1.995 (4)
Zn1—N1	2.061 (4)
Zn1—Cl2	2.3252 (18)
Zn1—Cl1	2.3628 (17)
Zn2—O3	1.994 (4)
Zn2—N3	2.095 (5)
Zn2—Cl3	2.2994 (16)
Zn2—Cl4	2.3575 (16)

**Table 2:** Hydrogen-bond geometry (Å, °)

*D*—H⋯*A*	*D*—H	H⋯*A*	*D*⋯*A*	*D*—H⋯*A*
N4—H4*B*⋯O1^i^	0.90	2.03	2.904 (6)	163
N4—H4*A*⋯Cl2^i^	0.90	2.73	3.466 (5)	140
N2—H2*B*⋯Cl3	0.90	2.74	3.484 (5)	140
N2—H2*A*⋯O3	0.90	2.03	2.892 (6)	161

Symmetry code: (i) 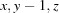.

**Fig. 1.**
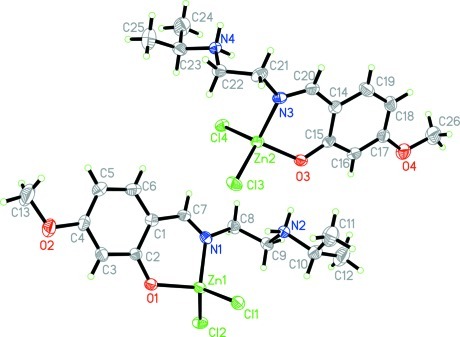
The molecular structure of the title compound with atom labels and 30% probability displacement ellipsoids for non-H atoms.

**Fig. 2.**
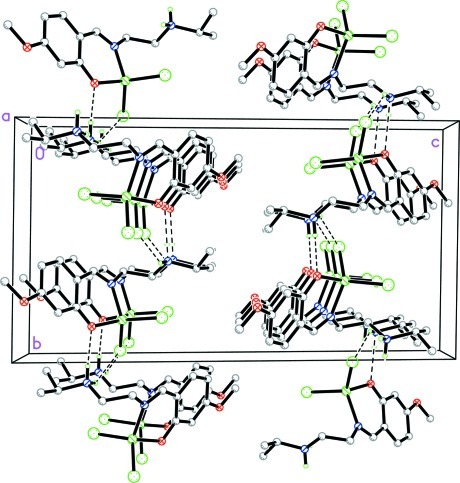
The molecular packing of the title compound, viewed down the a axis. Hydrogen bonds are drawn as dashed lines. H atoms not involved in hydrogen bonding have been omitted.

## supplementary crystallographic information

### Comment

The structures of zinc complexes with Schiff bases have received a great deal of attention (Munro *et al.*, 2009; Granifo *et al.*, 2006). The title compound was first reported in the monoclinic space group P2_1_/n (Han *et al.*, 2010). The author reports here a triclinic modification, I, in the space group P -1 with an asymmetric unit that contains two independent mononuclear zinc complex molecules (Fig. 1). In each molecule the Zn atoms are coordinated in a bidentate fashion by phenolate O and imine N atoms of the Schiff base ligand. Two Cl^-^ anions complete the tetrahedral coordination sphere in each case. The Zn1–O1, 1.995 (4)Å, Zn2–O3, 1.994 (4) Å, Zn1–N1, 2.061 (3)Å, Zn2–N3, 2.095 (3)Å, Zn1–Cl1, 2.3268, Zn1–Cl2, 2.3252 (18), Zn2–Cl3 2.2994 (16) and Zn2 Cl4 2.3575 (16) bond distances in the two independent molecules are comparable to one another but are generally longer than those observed in the monoclinic polymorph. This has Zn–O, 1.9425 (19) Å, Zn–N, 1.997 (2) Zn–Cl1 2.2554 (10) and Zn–Cl2, 2.2290 (9)Å. The bond lengths in I are also longer than those reported in other zinc Schiff base complexes (Ali *et al.*, 2008; Zhu, 2008; Wang, 2007). These differences may be due to packing effects. In the crystal, molecules are linked through N—H···O and N—H···Cl hydrogen bonds (Table 2), to form chains along the *b* axis (Fig. 2).

### Experimental

4-Methoxysalicylaldehyde (0.1 mmol, 15.2 mg), *N*-isopropylethane-1,2-diamine (0.1 mmol, 10.2 mg), and zinc chloride (0.1 mmol, 13.7 mg) were dissolved in methanol (15 ml). The mixture was stirred at reflux for 30 min and cooled to room temperature. Small and colorless block-like single crystals were obtained by slow evaporation of the solution in air.

### Refinement

Hydrogen atoms were positioned geometrically and refined using a riding model, with C—H = 0.93–0.97 Å, N—H = 0.90 Å, and with *U*_iso_(H) = 1.2*U*_eq_(C,*N*) and 1.5*U*_eq_(C_methyl_).

### Crystal data

**Table:**

[ZnCl_2_(C_13_H_20_N_2_O_2_)]	*Z* = 4
*M**_r_* = 372.58	*F*(000) = 768
Triclinic, *P*1	*D*_x_ = 1.362 Mg m^−^^3^
Hall symbol: -P 1	Mo *K*α radiation, λ = 0.71073 Å
*a* = 6.491 (3) Å	Cell parameters from 3080 reflections
*b* = 12.351 (2) Å	θ = 2.5–24.5°
*c* = 22.803 (3) Å	µ = 1.65 mm^−^^1^
α = 90.707 (2)°	*T* = 298 K
β = 96.201 (2)°	Block, colorless
γ = 90.660 (2)°	0.13 × 0.10 × 0.08 mm
*V* = 1817.1 (9) Å^3^	

### Data collection

**Table:**

Bruker SMART CCD area-detector diffractometer	5775 independent reflections
Radiation source: fine-focus sealed tube	4093 reflections with *I* > 2σ(*I*)
Graphite monochromator	*R*_int_ = 0.027
ω scans	θ_max_ = 25.0°, θ_min_ = 2.4°
Absorption correction: multi-scan (*SADABS*; Sheldrick, 2004)	*h* = −7→7
*T*_min_ = 0.814, *T*_max_ = 0.880	*k* = −14→14
8230 measured reflections	*l* = −27→24

### Refinement

**Table:**

Refinement on *F*^2^	Primary atom site location: structure-invariant direct methods
Least-squares matrix: full	Secondary atom site location: difference Fourier map
*R*[*F*^2^ > 2σ(*F*^2^)] = 0.055	Hydrogen site location: inferred from neighbouring sites
*wR*(*F*^2^) = 0.181	H-atom parameters constrained
*S* = 1.08	*w* = 1/[σ^2^(*F*_o_^2^) + (0.0975*P*)^2^ + 0.557*P*] where *P* = (*F*_o_^2^ + 2*F*_c_^2^)/3
5775 reflections	(Δ/σ)_max_ < 0.001
367 parameters	Δρ_max_ = 0.63 e Å^−^^3^
0 restraints	Δρ_min_ = −0.57 e Å^−^^3^

### Special details

**Table:**

Geometry. All e.s.d.'s (except the e.s.d. in the dihedral angle between two l.s. planes) are estimated using the full covariance matrix. The cell e.s.d.'s are taken into account individually in the estimation of e.s.d.'s in distances, angles and torsion angles; correlations between e.s.d.'s in cell parameters are only used when they are defined by crystal symmetry. An approximate (isotropic) treatment of cell e.s.d.'s is used for estimating e.s.d.'s involving l.s. planes.
Refinement. Refinement of *F*^2^ against ALL reflections. The weighted *R*-factor *wR* and goodness of fit *S* are based on *F*^2^, conventional *R*-factors *R* are based on *F*, with *F* set to zero for negative *F*^2^. The threshold expression of *F*^2^ > σ(*F*^2^) is used only for calculating *R*-factors(gt) *etc*. and is not relevant to the choice of reflections for refinement. *R*-factors based on *F*^2^ are statistically about twice as large as those based on *F*, and *R*- factors based on ALL data will be even larger.

### Fractional atomic coordinates and isotropic or equivalent isotropic displacement parameters (Å^2^)

**Table:**

	*x*	*y*	*z*	*U*_iso_*/*U*_eq_	
Zn1	0.74321 (12)	0.83418 (5)	0.23642 (3)	0.0441 (2)	
Cl1	0.9409 (3)	0.81988 (11)	0.32920 (7)	0.0474 (4)	
Cl2	0.4987 (2)	0.97077 (12)	0.22753 (8)	0.0521 (4)	
O1	0.9277 (7)	0.8554 (3)	0.17284 (19)	0.0498 (11)	
O2	1.4088 (9)	0.7419 (4)	0.0412 (2)	0.0766 (16)	
N1	0.6280 (7)	0.6838 (4)	0.2085 (2)	0.0397 (12)	
N2	0.5954 (8)	0.5896 (3)	0.3369 (2)	0.0392 (11)	
H2A	0.5568	0.5194	0.3339	0.047*	
H2B	0.7167	0.5959	0.3214	0.047*	
C1	0.8974 (9)	0.6630 (4)	0.1382 (3)	0.0423 (14)	
C2	0.9929 (9)	0.7736 (4)	0.1399 (2)	0.0396 (14)	
C3	1.1644 (10)	0.7914 (5)	0.1059 (3)	0.0481 (16)	
H3	1.2275	0.8595	0.1070	0.058*	
C4	1.2390 (11)	0.7097 (5)	0.0713 (3)	0.0508 (16)	
C5	1.1530 (13)	0.6025 (6)	0.0688 (3)	0.065 (2)	
H5	1.2054	0.5479	0.0465	0.078*	
C6	0.9840 (12)	0.5833 (5)	0.1021 (3)	0.063 (2)	
H6	0.9252	0.5142	0.1003	0.076*	
C7	0.7202 (10)	0.6298 (5)	0.1696 (3)	0.0448 (15)	
H7	0.6669	0.5608	0.1604	0.054*	
C8	0.4381 (10)	0.6345 (5)	0.2314 (3)	0.0523 (17)	
H8A	0.4358	0.5571	0.2238	0.063*	
H8B	0.3149	0.6645	0.2101	0.063*	
C9	0.4320 (10)	0.6553 (5)	0.2982 (3)	0.0443 (15)	
H9A	0.2951	0.6364	0.3086	0.053*	
H9B	0.4555	0.7318	0.3067	0.053*	
C10	0.6342 (12)	0.6199 (6)	0.4030 (3)	0.0606 (19)	
H10	0.6890	0.6942	0.4057	0.073*	
C11	0.8045 (14)	0.5466 (7)	0.4341 (4)	0.088 (3)	
H11A	0.7653	0.4720	0.4274	0.131*	
H11B	0.8198	0.5621	0.4758	0.131*	
H11C	0.9336	0.5606	0.4185	0.131*	
C12	0.4325 (15)	0.6195 (8)	0.4329 (4)	0.095 (3)	
H12A	0.3364	0.6690	0.4131	0.142*	
H12B	0.4624	0.6413	0.4735	0.142*	
H12C	0.3728	0.5478	0.4306	0.142*	
C13	1.4981 (15)	0.6614 (8)	0.0035 (4)	0.097 (3)	
H13A	1.5361	0.5983	0.0262	0.146*	
H13B	1.6189	0.6918	−0.0112	0.146*	
H13C	1.3977	0.6416	−0.0290	0.146*	
Zn2	0.75666 (12)	0.33416 (5)	0.26356 (3)	0.0443 (2)	
Cl3	1.0020 (2)	0.47085 (11)	0.27261 (8)	0.0523 (4)	
Cl4	0.5584 (2)	0.31981 (11)	0.17081 (7)	0.0475 (4)	
O3	0.5732 (7)	0.3560 (3)	0.32735 (19)	0.0482 (11)	
O4	0.0905 (9)	0.2412 (4)	0.4588 (2)	0.0797 (17)	
N3	0.8731 (7)	0.1838 (3)	0.2917 (2)	0.0390 (12)	
N4	0.9042 (8)	0.0893 (4)	0.1632 (2)	0.0422 (12)	
H4A	0.7834	0.0954	0.1789	0.051*	
H4B	0.9398	0.0191	0.1652	0.051*	
C14	0.6016 (10)	0.1639 (5)	0.3615 (3)	0.0453 (15)	
C15	0.5066 (9)	0.2740 (4)	0.3594 (3)	0.0415 (15)	
C16	0.3387 (10)	0.2910 (5)	0.3940 (3)	0.0495 (16)	
H16	0.2789	0.3591	0.3939	0.059*	
C17	0.2592 (11)	0.2094 (5)	0.4285 (3)	0.0544 (18)	
C18	0.3474 (12)	0.1040 (6)	0.4310 (3)	0.061 (2)	
H18	0.2961	0.0503	0.4539	0.074*	
C19	0.5152 (12)	0.0830 (5)	0.3975 (3)	0.063 (2)	
H19	0.5722	0.0142	0.3989	0.075*	
C20	0.7798 (10)	0.1295 (5)	0.3312 (3)	0.0454 (16)	
H20	0.8328	0.0616	0.3409	0.054*	
C21	1.0593 (10)	0.1347 (5)	0.2682 (3)	0.0521 (17)	
H21A	1.0568	0.0572	0.2745	0.063*	
H21B	1.1838	0.1641	0.2904	0.063*	
C22	1.0700 (10)	0.1552 (5)	0.2021 (3)	0.0443 (15)	
H22A	1.0505	0.2317	0.1945	0.053*	
H22B	1.2062	0.1360	0.1919	0.053*	
C23	0.8673 (12)	0.1201 (5)	0.0974 (3)	0.0581 (18)	
H23	0.8154	0.1943	0.0958	0.070*	
C24	0.6975 (15)	0.0471 (7)	0.0662 (4)	0.088 (3)	
H24A	0.7425	−0.0267	0.0676	0.132*	
H24B	0.6691	0.0685	0.0259	0.132*	
H24C	0.5740	0.0535	0.0856	0.132*	
C25	1.0646 (15)	0.1192 (8)	0.0672 (4)	0.092 (3)	
H25A	1.1609	0.1720	0.0858	0.138*	
H25B	1.0327	0.1364	0.0263	0.138*	
H25C	1.1251	0.0487	0.0705	0.138*	
C26	0.0028 (15)	0.1620 (7)	0.4963 (4)	0.093 (3)	
H26A	−0.0352	0.0973	0.4738	0.140*	
H26B	−0.1179	0.1915	0.5112	0.140*	
H26C	0.1036	0.1450	0.5288	0.140*	

### Atomic displacement parameters (Å^2^)

**Table:**

	*U*^11^	*U*^22^	*U*^33^	*U*^12^	*U*^13^	*U*^23^
Zn1	0.0476 (5)	0.0345 (4)	0.0517 (5)	−0.0044 (3)	0.0124 (4)	−0.0003 (3)
Cl1	0.0494 (10)	0.0459 (8)	0.0460 (9)	−0.0042 (7)	0.0015 (7)	0.0037 (6)
Cl2	0.0399 (10)	0.0434 (8)	0.0735 (11)	0.0052 (7)	0.0088 (8)	−0.0091 (7)
O1	0.055 (3)	0.036 (2)	0.062 (3)	−0.0052 (19)	0.025 (2)	−0.0056 (19)
O2	0.091 (4)	0.074 (3)	0.073 (4)	0.014 (3)	0.045 (3)	0.006 (3)
N1	0.035 (3)	0.040 (3)	0.044 (3)	−0.003 (2)	0.001 (2)	0.006 (2)
N2	0.041 (3)	0.037 (2)	0.040 (3)	−0.001 (2)	0.005 (2)	0.001 (2)
C1	0.038 (4)	0.042 (3)	0.046 (3)	0.000 (3)	0.000 (3)	0.000 (3)
C2	0.046 (4)	0.035 (3)	0.037 (3)	0.006 (3)	0.000 (3)	0.006 (2)
C3	0.048 (4)	0.046 (3)	0.052 (4)	0.002 (3)	0.011 (3)	0.008 (3)
C4	0.057 (5)	0.060 (4)	0.039 (4)	0.019 (3)	0.015 (3)	0.009 (3)
C5	0.079 (6)	0.055 (4)	0.066 (5)	0.008 (4)	0.028 (4)	−0.010 (3)
C6	0.075 (5)	0.044 (4)	0.071 (5)	−0.005 (3)	0.012 (4)	−0.013 (3)
C7	0.048 (4)	0.036 (3)	0.050 (4)	−0.007 (3)	0.004 (3)	−0.005 (3)
C8	0.050 (4)	0.057 (4)	0.049 (4)	−0.016 (3)	0.003 (3)	0.004 (3)
C9	0.035 (4)	0.042 (3)	0.057 (4)	0.001 (3)	0.010 (3)	0.007 (3)
C10	0.070 (5)	0.059 (4)	0.053 (4)	−0.011 (4)	0.011 (4)	−0.009 (3)
C11	0.092 (7)	0.110 (7)	0.058 (5)	0.004 (5)	−0.005 (5)	0.001 (5)
C12	0.092 (7)	0.123 (8)	0.073 (6)	−0.013 (6)	0.033 (5)	−0.025 (5)
C13	0.105 (8)	0.115 (7)	0.082 (7)	0.027 (6)	0.052 (6)	0.003 (5)
Zn2	0.0480 (5)	0.0349 (4)	0.0518 (5)	−0.0010 (3)	0.0141 (4)	−0.0034 (3)
Cl3	0.0424 (10)	0.0413 (8)	0.0741 (11)	−0.0101 (7)	0.0106 (8)	0.0054 (7)
Cl4	0.0507 (10)	0.0458 (8)	0.0448 (9)	0.0017 (7)	0.0011 (7)	−0.0079 (6)
O3	0.054 (3)	0.034 (2)	0.060 (3)	−0.0038 (19)	0.024 (2)	−0.0033 (19)
O4	0.092 (4)	0.076 (3)	0.079 (4)	−0.016 (3)	0.049 (3)	−0.009 (3)
N3	0.034 (3)	0.036 (2)	0.045 (3)	0.005 (2)	0.000 (2)	−0.006 (2)
N4	0.051 (3)	0.037 (2)	0.040 (3)	−0.004 (2)	0.010 (2)	−0.004 (2)
C14	0.043 (4)	0.047 (3)	0.046 (4)	−0.004 (3)	0.007 (3)	−0.004 (3)
C15	0.036 (4)	0.045 (3)	0.043 (3)	−0.007 (3)	0.003 (3)	−0.014 (3)
C16	0.050 (4)	0.046 (3)	0.053 (4)	−0.001 (3)	0.007 (3)	−0.010 (3)
C17	0.065 (5)	0.052 (4)	0.048 (4)	−0.017 (3)	0.012 (3)	−0.007 (3)
C18	0.070 (5)	0.063 (4)	0.055 (4)	−0.016 (4)	0.023 (4)	0.006 (3)
C19	0.073 (5)	0.046 (4)	0.070 (5)	−0.004 (3)	0.008 (4)	0.010 (3)
C20	0.051 (4)	0.038 (3)	0.046 (4)	0.001 (3)	0.000 (3)	−0.004 (3)
C21	0.043 (4)	0.058 (4)	0.053 (4)	−0.002 (3)	0.001 (3)	−0.019 (3)
C22	0.037 (4)	0.040 (3)	0.058 (4)	0.000 (3)	0.013 (3)	−0.004 (3)
C23	0.073 (5)	0.047 (4)	0.054 (4)	0.003 (3)	0.003 (4)	0.005 (3)
C24	0.103 (7)	0.101 (6)	0.057 (5)	−0.015 (5)	−0.006 (5)	−0.009 (4)
C25	0.096 (7)	0.123 (8)	0.061 (5)	0.000 (6)	0.025 (5)	0.012 (5)
C26	0.096 (8)	0.101 (7)	0.088 (7)	−0.029 (6)	0.044 (6)	0.000 (5)

### Geometric parameters (Å, º)

**Table:**

Zn1—O1	1.995 (4)	Zn2—O3	1.994 (4)
Zn1—N1	2.061 (4)	Zn2—N3	2.095 (5)
Zn1—Cl2	2.3252 (18)	Zn2—Cl3	2.2994 (16)
Zn1—Cl1	2.3628 (17)	Zn2—Cl4	2.3575 (16)
O1—C2	1.351 (6)	O3—C15	1.350 (7)
O2—C4	1.416 (8)	O4—C17	1.414 (8)
O2—C13	1.471 (9)	O4—C26	1.458 (8)
N1—C7	1.304 (7)	N3—C20	1.323 (7)
N1—C8	1.514 (7)	N3—C21	1.505 (8)
N2—C10	1.543 (8)	N4—C22	1.537 (7)
N2—C9	1.547 (7)	N4—C23	1.548 (8)
N2—H2A	0.9000	N4—H4A	0.9000
N2—H2B	0.8999	N4—H4B	0.9000
C1—C6	1.433 (9)	C14—C19	1.448 (9)
C1—C7	1.475 (8)	C14—C20	1.474 (9)
C1—C2	1.492 (7)	C14—C15	1.498 (8)
C2—C3	1.440 (8)	C15—C16	1.430 (8)
C3—C4	1.395 (8)	C16—C17	1.412 (9)
C3—H3	0.9300	C16—H16	0.9300
C4—C5	1.429 (9)	C17—C18	1.428 (10)
C5—C6	1.419 (10)	C18—C19	1.420 (10)
C5—H5	0.9300	C18—H18	0.9300
C6—H6	0.9300	C19—H19	0.9300
C7—H7	0.9300	C20—H20	0.9300
C8—C9	1.546 (9)	C21—C22	1.540 (9)
C8—H8A	0.9700	C21—H21A	0.9700
C8—H8B	0.9700	C21—H21B	0.9700
C9—H9A	0.9700	C22—H22A	0.9700
C9—H9B	0.9700	C22—H22B	0.9700
C10—C12	1.539 (11)	C23—C25	1.518 (11)
C10—C11	1.553 (11)	C23—C24	1.524 (10)
C10—H10	0.9800	C23—H23	0.9800
C11—H11A	0.9600	C24—H24A	0.9600
C11—H11B	0.9600	C24—H24B	0.9600
C11—H11C	0.9600	C24—H24C	0.9600
C12—H12A	0.9600	C25—H25A	0.9600
C12—H12B	0.9600	C25—H25B	0.9600
C12—H12C	0.9600	C25—H25C	0.9600
C13—H13A	0.9600	C26—H26A	0.9600
C13—H13B	0.9600	C26—H26B	0.9600
C13—H13C	0.9600	C26—H26C	0.9600
			
O1—Zn1—N1	96.96 (18)	O3—Zn2—N3	96.87 (18)
O1—Zn1—Cl2	107.23 (13)	O3—Zn2—Cl3	107.48 (12)
N1—Zn1—Cl2	114.06 (15)	N3—Zn2—Cl3	113.43 (14)
O1—Zn1—Cl1	110.63 (14)	O3—Zn2—Cl4	110.55 (14)
N1—Zn1—Cl1	109.73 (14)	N3—Zn2—Cl4	111.53 (13)
Cl2—Zn1—Cl1	116.43 (6)	Cl3—Zn2—Cl4	115.33 (6)
C2—O1—Zn1	123.6 (3)	C15—O3—Zn2	123.1 (3)
C4—O2—C13	118.2 (6)	C17—O4—C26	118.0 (6)
C7—N1—C8	118.9 (5)	C20—N3—C21	118.6 (5)
C7—N1—Zn1	119.1 (4)	C20—N3—Zn2	119.2 (4)
C8—N1—Zn1	122.0 (4)	C21—N3—Zn2	122.2 (4)
C10—N2—C9	117.6 (5)	C22—N4—C23	116.8 (4)
C10—N2—H2A	107.9	C22—N4—H4A	108.1
C9—N2—H2A	107.8	C23—N4—H4A	108.2
C10—N2—H2B	107.9	C22—N4—H4B	108.1
C9—N2—H2B	107.9	C23—N4—H4B	108.1
H2A—N2—H2B	107.2	H4A—N4—H4B	107.3
C6—C1—C7	117.8 (5)	C19—C14—C20	115.8 (6)
C6—C1—C2	116.7 (6)	C19—C14—C15	117.8 (6)
C7—C1—C2	125.5 (5)	C20—C14—C15	126.3 (5)
O1—C2—C3	119.7 (5)	O3—C15—C16	119.6 (5)
O1—C2—C1	122.5 (5)	O3—C15—C14	123.4 (5)
C3—C2—C1	117.8 (5)	C16—C15—C14	117.0 (5)
C4—C3—C2	122.1 (6)	C17—C16—C15	123.2 (6)
C4—C3—H3	118.9	C17—C16—H16	118.4
C2—C3—H3	118.9	C15—C16—H16	118.4
C3—C4—O2	114.2 (6)	C16—C17—O4	114.8 (6)
C3—C4—C5	121.9 (6)	C16—C17—C18	120.7 (6)
O2—C4—C5	123.9 (6)	O4—C17—C18	124.5 (6)
C6—C5—C4	116.9 (6)	C19—C18—C17	118.4 (6)
C6—C5—H5	121.6	C19—C18—H18	120.8
C4—C5—H5	121.6	C17—C18—H18	120.8
C5—C6—C1	124.6 (6)	C18—C19—C14	122.9 (6)
C5—C6—H6	117.7	C18—C19—H19	118.5
C1—C6—H6	117.7	C14—C19—H19	118.5
N1—C7—C1	129.1 (5)	N3—C20—C14	127.6 (6)
N1—C7—H7	115.5	N3—C20—H20	116.2
C1—C7—H7	115.5	C14—C20—H20	116.2
N1—C8—C9	112.8 (5)	N3—C21—C22	113.5 (5)
N1—C8—H8A	109.0	N3—C21—H21A	108.9
C9—C8—H8A	109.0	C22—C21—H21A	108.9
N1—C8—H8B	109.0	N3—C21—H21B	108.9
C9—C8—H8B	109.0	C22—C21—H21B	108.9
H8A—C8—H8B	107.8	H21A—C21—H21B	107.7
C8—C9—N2	112.9 (5)	N4—C22—C21	111.8 (5)
C8—C9—H9A	109.0	N4—C22—H22A	109.3
N2—C9—H9A	109.0	C21—C22—H22A	109.3
C8—C9—H9B	109.0	N4—C22—H22B	109.3
N2—C9—H9B	109.0	C21—C22—H22B	109.3
H9A—C9—H9B	107.8	H22A—C22—H22B	107.9
C12—C10—N2	112.0 (6)	C25—C23—C24	112.7 (6)
C12—C10—C11	113.4 (7)	C25—C23—N4	112.8 (6)
N2—C10—C11	110.1 (6)	C24—C23—N4	109.3 (6)
C12—C10—H10	107.0	C25—C23—H23	107.3
N2—C10—H10	107.0	C24—C23—H23	107.3
C11—C10—H10	107.0	N4—C23—H23	107.3
C10—C11—H11A	109.5	C23—C24—H24A	109.5
C10—C11—H11B	109.5	C23—C24—H24B	109.5
H11A—C11—H11B	109.5	H24A—C24—H24B	109.5
C10—C11—H11C	109.5	C23—C24—H24C	109.5
H11A—C11—H11C	109.5	H24A—C24—H24C	109.5
H11B—C11—H11C	109.5	H24B—C24—H24C	109.5
C10—C12—H12A	109.5	C23—C25—H25A	109.5
C10—C12—H12B	109.5	C23—C25—H25B	109.5
H12A—C12—H12B	109.5	H25A—C25—H25B	109.5
C10—C12—H12C	109.5	C23—C25—H25C	109.5
H12A—C12—H12C	109.5	H25A—C25—H25C	109.5
H12B—C12—H12C	109.5	H25B—C25—H25C	109.5
O2—C13—H13A	109.5	O4—C26—H26A	109.5
O2—C13—H13B	109.5	O4—C26—H26B	109.5
H13A—C13—H13B	109.5	H26A—C26—H26B	109.5
O2—C13—H13C	109.5	O4—C26—H26C	109.5
H13A—C13—H13C	109.5	H26A—C26—H26C	109.5
H13B—C13—H13C	109.5	H26B—C26—H26C	109.5

### Hydrogen-bond geometry (Å, º)

**Table:**

*D*—H···*A*	*D*—H	H···*A*	*D*···*A*	*D*—H···*A*
N4—H4*B*···O1^i^	0.90	2.03	2.904 (6)	163
N4—H4*A*···Cl2^i^	0.90	2.73	3.466 (5)	140
N2—H2*B*···Cl3	0.90	2.74	3.484 (5)	140
N2—H2*A*···O3	0.90	2.03	2.892 (6)	161

Symmetry code: (i) *x*, *y*−1, *z*.
